# Social engagement pattern, health behaviors and subjective well-being of older adults: an international perspective using WHO-SAGE survey data

**DOI:** 10.1186/s12889-019-7841-7

**Published:** 2020-01-23

**Authors:** Mengyun Luo, Ding Ding, Adrian Bauman, Joel Negin, Philayrath Phongsavan

**Affiliations:** 1 The University of Sydney, Sydney School of Public Health, Faculty of Medicine and Health and Charles Perkins Centre, Sydney, NSW 2006 Australia; 2 School of Public Health, School of Medicine, Shanghai Jiao Tong University, Shanghai, 200025 People’s Republic of China

**Keywords:** Social engagement, Health behavior, Lifestyle, Subjective well-being, Low- to middle-income countries, Ageing

## Abstract

**Background:**

Social engagement forms the basis of social relationships by providing a sense of belonging, social identity, and fulfillment. Previous research demonstrates that social engagement was associated with positive health behaviors among older adults. However, the results have been different across health-related behaviors, and mostly based on data from high-income countries. For example, studies from the US and UK showed that social engagement was protective against smoking, while others found social engagement encouraged more smoking in many Asian cultures. In this study, we aim to examine the association between social engagement and a range of health-related behaviors and subjective well-being among older adults in six low- to middle-income countries.

**Methods:**

Data from the WHO Study on Global Ageing and Adult Health (SAGE Wave 1) were used. A total of 33,338 individuals aged 50 and older in China, Russia, India, Ghana, South Africa, and Mexico were included. Social engagement, tobacco use, alcohol consumption, fruit and vegetable intake, physical activity, sedentary behavior, sleep duration, depression symptoms, self-rated health status, and quality of life were assessed using established self-reported measures. Multiple logistic regression models were used to examine the relationship between social engagement and nine outcome variables, adjusting for socio-demographic characteristics.

**Results:**

Lower levels of social engagement were positively related to physical inactivity, prolonged sitting time, unhealthy sleep duration, perceived depression, poor self-rated health, and low quality of life. However, the associations between social engagement and tobacco use, excessive drinking, and insufficient fruit and vegetable intake were mixed across countries.

**Conclusion:**

This international study found high social engagement as a potential health-promoting factor in some low- to middle-income countries. Although the impacts of social engagement on tobacco and alcohol use and diet were complicated and culture-specific, interventions at both individual and community levels should encourage healthy lifestyles through positive social engagement.

## Background

WHO defines healthy aging as the process of developing and maintaining the functional ability that enables wellbeing in older age [1]. One important component of functional ability is to build and maintain relationships, as well as contribute to society, which means to engage in both individual- and society-level activities. Social engagement, also called social participation or social involvement, forms the basis of social relationships or participation in a community, and provides a sense of belonging, social identity, and fulfillment. Evidence from cross-sectional studies among older adults suggests positive associations of socially meaningful relations with mental well-being and quality of life, and inverse associations with depressive symptoms [2, 3]. In addition, several longitudinal studies have shown that social engagement was associated with a lower risk of heart disease, cancers and all-cause mortality [4, 5].

Social engagement has been found to be associated with positive health behaviors among older adults [6]. For example, higher levels of social engagement were reported to be positively associated with consuming at least five daily servings of fruit and vegetable and moderate-to-vigorous physical activity [7, 8]. Kawachi and Berkman suggested that engaging in social activities promoted healthy behaviors and discouraged unhealthy ones by enhancing psychosocial processes through the provision of emotional support from trusted social networks, such as family, friends, neighbors and community [9].

However, the results so far have differed across health-related lifestyle behaviors. For example, Hiroyuki et al. showed that those with higher social engagement spent approximately 10–20% less time on leisure time sedentary behaviors [8], while others reported inconsistent findings or no associations [10]. Lindstrom and Samuel et al. showed that social engagement was protective against smoking in the US and UK [7, 11], while Sapag et al. [12] found that networks encouraged more smoking in many Asian cultures, where social norms supported smoking in social settings [13]. Similarly, social engagement was found to facilitate alcohol consumption among older Australians [14]. These findings suggest that the relationships between social engagement and health behaviors may differ by culture and by health behavior. To date, most studies on social engagement and health behavior are based on data from high-income countries (HICs). Therefore, comparison across multiple health behaviors using established measures across multiple low- to middle-income countries (LMICs) may fill an important knowledge gap by shedding light on the role that social engagement plays in health.

In this study, we examined the association between social engagement and a range of health-related lifestyle behaviors (i.e., tobacco and alcohol consumption, fruit and vegetable intake, physical activity, sedentary behavior and sleep duration) and subjective well-being (i.e., depression, self-rated health, and quality of life), among older adults in six LMICs. Having a better understanding of how these relationships are patterned could help inform policy and public health actions within a LMIC context.

## Methods

### Study design

This study used nationally representative data from the World Health Organization’s Study on Global Ageing and Adult Health (WHO SAGE) Wave 1, which was carried out in six LMICs (China, Russia, India, Ghana, South Africa and Mexico) from 2007 to 2010. Although SAGE is a longitudinal study and a baseline survey (SAGE Wave 0) was created during 2002–2004, only Wave 1 data were used in this study because China and South Africa did not follow up Wave 0 respondents for Wave 1. China, Russia, India, Ghana and South Africa all used stratified multi-stage clustered sampling strategy, and Mexico included extra supplementary and replacement samples in Wave 1 to account for losses to follow up since Wave 0 [15]. SAGE was approved by the Ethics Review Committee of the World Health Organization.

### Participants

WHO SAGE (Wave 1) focused on older adults aged 50+ years old (*n* = 13,367 in China, 3938 in Russia, 7150 in India, 4724 in Ghana, 3840 in South Africa, 2315 in Mexico). Participants with proxy answers, cognitive limitations, implausible answers in physical activity, invalid answers in social engagement, and missing or zero value for post-stratified individual probability weights were removed from the analysis. The final sample sizes included in this analysis were: 12,837 for China, 3682 for Russia, 6551 for India, 4273 for Ghana, 3609 for South Africa, and 2193 for Mexico.

### Exposure variable

The 9 social engagement questions asked of participants in all six countries are listed in Appendix 1. These questions asked about the frequency of several social activities in the previous 12 months, which had been used before and reported elsewhere [16]. The response options were rated on a 5-point Likert scale ranging from 1 (never) to 5 (daily). Scores from these questions were summed and divided by 9 to derive a mean social engagement score for India, Ghana, South Africa and Mexico. Due to the low prevalence of religious practices in China and Russia, only 8 items (excluding the religious service question) were used for these two countries. For China and Russia, the summed score from the 8 questions was divided by 8, the total number of questions used in these two countries. Social engagement was used as a continuous variable in this study, with higher scores representing higher levels of social engagement. The Cronbach’s alpha internal consistency coefficients for these groups of social engagement items ranged from 0.63 to 0.81 across six countries. (See more details in Appendix 1).

### Outcome variables

#### Tobacco and alcohol use and diet

“Tobacco use” was defined as currently smoking tobacco or using any tobacco products. Harmful drinking was defined as consuming 14 or more standard drinks per week for both men and women. Information on fruit and vegetable intake was based on the number of servings on a typical day. Less than five servings per day were considered insufficient [17].

#### Activity-related behaviors

Physical inactivity was measured by the Global Physical Activity Questionnaire (GPAQ), which collected information about physical activity in three domains (activity at work (paid/unpaid), travel to and from places, recreational activities) and sedentary behavior [18]. This questionnaire is the standard physical activity measure for population surveillance in the WHO STEPS program [19].

Sedentary behavior was assessed using a single item derived from GPAQ about the time usually spent sitting or reclining on a typical day. This included sitting at a desk, sitting with friends, travelling in car, bus, train, reading, playing cards or watching television. More than 7 hours/day was regarded as prolonged sitting time [20].

Sleep duration was self-reported by the participants, which was comparable with single-item instruments used by previous studies. Either less than 7 hours/day or more than 9 hours/day was considered unhealthy [21, 22]. As Mexico did not collect any information about sleep duration, this outcome variable was not considered for Mexico.

#### Subjective well-being

Perceived depression symptom was assessed by one question, “Overall in the last 30 days, how much of a problem did you have with feeling sad, low or depressed?” [23] The answer ranged from 1 (none) to 5 (extreme). Those who responded with “none” were classified as not having perceived depression, while all other answers were regarded as having perceived depression.

Self-rated health status was measured by one question, “In general, how would you rate your health today?” The answer ranged from 1 (very good) to 5 (very bad). Those who answered 4 or 5 were classified as “poor self-rated health”.

Quality of life was assessed using EUROHIS-QOL 8-item index, a shortened version of the World Health Organization Quality of Life Instrument-Abbreviated Version (WHOQOL-BREF) and had acceptable reliability and validity [24]. Response to each question was based on a 5-point likert scale. A binary categorization of the EUROHIS-QOL was created as low quality of life (range 0–3) and high quality of life (range 3.01–5); this was based on mean score dichotomization for the total score (Cronbach’s α > 0.8 for all countries).

#### Covariates

Sociodemographic information including sex, age, marital status, education level, place of residence (urban/rural), country-specific income quintiles and working status were treated as covariates in this study. Age was categorized as 50–59, 60–69, 70–79, and 80 years and older.

#### Statistical analysis

To ensure the representativeness of the sample and adjusting for the complex survey sampling design, post-stratified individual non-zero probability weights, and country-specific strata and clustering were used for all statistical analysis in each country. Social engagement scores were presented as means and standard errors, while the sociodemographic information was reported as weighted percentages. We used forced entry logistic regression models to examine the association between social engagement and each outcome variable separately in each country. Multiple logistic regression models were used following univariate models to examine the association between social engagement and the outcome variables after controlling for sex, age, marital status, education level, place of residence, income quintiles, and working status. All analyses were performed with complex samples analysis using SPSS Version 24.0. The significant level was set at 0.05.

## Results

Weighted sample characteristics are presented in Table 1. A total of 33,145 individuals in the six countries were included in this analysis. China had the largest sample (*n* = 12,837), while Mexico had the smallest (*n* = 2193). The distributions of the sociodemographic characteristics varied across six countries. Over half of the sample were females except in India and Ghana. All six countries had the highest proportion of participants in the age group 50–59 years. The majority were currently married or cohabiting in all six countries. India and Ghana had the lowest education level, with over 50% reporting no schooling. In contrast, half of the people in Russia had completed high school or equivalent and around one fifth had a college degree or above. Most of the samples in Russia, South Africa and Mexico lived in urban areas, while more than half of the participants lived in rural areas in India, Ghana and China. Ghana had the highest proportion of people who were currently working, while South Africa had the lowest proportion.

**Table 1:** Weighted sample characteristics across six countries

Socio-demographics	China (*n* = 12,837) %	Russia (*n* = 3682) %	India (*n* = 6551) %	Ghana (*n* = 4273) %	South Africa (*n* = 3609) %	Mexico (*N* = 2193) %
Sex						
Male	49.8	39.1	51.0	52.3	43.9	46.7
Female	50.2	60.9	49.0	47.7	56.1	53.3
Age group						
50–59 years	45.2	45.2	48.6	39.8	49.6	49.1
60–69 years	32.0	24.7	30.9	27.5	31.0	25.7
70–79 years	18.5	21.6	16.0	23.1	13.9	17.9
≥ 80 years	4.3	8.5	4.5	9.6	5.5	7.3
Marital status						
Never married/ Separated/ Divorced/ Widowed	14.6	41.6	23.1	40.8	44.0	27.1
Currently married/ Cohabiting	85.4	58.4	76.9	59.2	56.0	72.9
Education level						
Never been to school	22.3	0.7	51.2	54.0	23.6	17.3
Secondary school and lower	60.4	26.9	35.0	25.3	61.9	72.2
High school (or equivalent) completed	12.8	54.2	8.6	17.2	8.7	2.4
College and above	4.6	18.3	5.1	3.6	5.8	8.1
Place of residence						
Urban	47.7	72.7	28.9	41.1	65.2	78.4
Rural	52.3	27.3	71.1	58.9	34.8	21.6
Income quintiles (within country)						
Lowest	16.1	16.1	18.2	18.3	20.0	15.3
Second	18.0	19.6	19.5	19.1	20.0	25.0
Middle	20.3	19.0	18.8	20.6	18.8	16.4
Fourth	23.6	20.8	19.6	20.6	20.0	16.6
Highest	22.0	24.4	23.9	21.4	21.2	26.7
Working status						
Not working	56.4	59.8	56.9	30.9	69.7	62.6
Still working	43.6	40.2	43.1	69.1	30.3	37.4

Note: Weighted estimates

### Social engagement scores

Table 2 shows the mean score of social engagement across six countries stratified by sociodemographic variables. Ghana had the highest total mean score, while Mexico had the lowest. Males, those who are currently married or cohabiting, those living in rural areas, those younger and better educated were more likely to have higher social engagement scores. Those with higher income levels also reported higher social engagement, except for Ghana, where the middle-income quintile group had the highest level of social engagement, and South Africa, where the second-income quintile group had the highest mean score. People who were still working had a higher mean score than those who were not working in all six countries.

**Table 2:** Mean social engagement score by socio-demographic characteristics across six countries, Mean (Standard Error)

	China	Russia	India	Ghana	South Africa	Mexico
Total	1.76 (0.01)	1.77 (0.02)	1.97 (0.02)	2.68 (0.02)	2.37 (0.02)	1.68 (0.04)
Sex						
Male	1.78 (0.01)	1.79 (0.04)	2.16 (0.02)	2.78 (0.03)	2.41 (0.03)	1.72 (0.07)
Female	1.73 (0.02)	1.75 (0.02)	1.76 (0.02)	2.56 (0.02)	2.34 (0.03)	1.64 (0.04)
Age group						
50–59	1.81 (0.01)	1.92 (0.04)	2.05 (0.02)	2.81 (0.03)	2.44 (0.03)	1.67 (0.08)
60–69	1.76 (0.01)	1.75 (0.02)	1.94 (0.03)	2.69 (0.03)	2.36 (0.03)	1.73 (0.05)
70–79	1.67 (0.02)	1.62 (0.04)	1.85 (0.02)	2.55 (0.03)	2.28 (0.04)	1.66 (0.04)
≥ 80	1.52 (0.03)	1.39 (0.03)	1.67 (0.04)	2.38 (0.06)	2.03 (0.07)	1.59 (0.05)
Marital status						
Never married/ Separated/ Divorced/ Widowed	1.67 (0.02)	1.69 (0.03)	1.73 (0.03)	2.53 (0.03)	2.32 (0.03)	1.60 (0.04)
Currently married/ Cohabiting	1.77 (0.01)	1.82 (0.02)	2.04 (0.02)	2.78 (0.02)	2.41 (0.03)	1.71 (0.06)
Education level						
Never been to school	1.67 (0.02)	1.35 (0.05)	1.84 (0.02)	2.58 (0.03)	2.32 (0.04)	1.57 (0.05)
Secondary school and lower	1.78 (0.01)	1.61 (0.05)	2.03 (0.03)	2.82 (0.04)	2.40 (0.02)	1.66 (0.05)
High school (or equivalent) completed	1.80 (0.03)	1.78 (0.03)	2.23 (0.04)	2.74 (0.04)	2.31 (0.09)	1.81 (0.18)
College and above	1.78 (0.05)	2.00 (0.06)	2.41 (0.07)	2.88 (0.06)	2.41 (0.13)	2.03 (0.13)
Place of residence						
Urban	1.69 (0.02)	1.77 (0.02)	1.91 (0.05)	2.55 (0.03)	2.35 (0.03)	1.68 (0.05)
Rural	1.81 (0.01)	1.76 (0.04)	1.99 (0.02)	2.77 (0.03)	2.40 (0.03)	1.68 (0.11)
Income quintiles						
Lowest	1.63 (0.02)	1.61 (0.04)	1.83 (0.03)	2.52 (0.04)	2.35 (0.05)	1.66 (0.05)
Second	1.74 (0.01)	1.73 (0.04)	1.89 (0.02)	2.68 (0.04)	2.40 (0.04)	1.43 (0.05)
Middle	1.78 (0.02)	1.69 (0.05)	1.96 (0.03)	2.78 (0.04)	2.39 (0.04)	1.79 (0.11)
Fourth	1.79 (0.02)	1.85 (0.03)	2.03 (0.03)	2.75 (0.03)	2.34 (0.05)	1.61 (0.04)
Highest	1.80 (0.03)	1.90 (0.06)	2.09 (0.03)	2.64 (0.04)	2.37 (0.05)	1.89 (0.08)
Working status						
Not working	1.68 (0.02)	1.63 (0.03)	1.80 (0.02)	2.26 (0.03)	2.26 (0.02)	1.65 (0.04)
Still working	1.86 (0.01)	1.98 (0.04)	2.19 (0.02)	2.86 (0.02)	2.62 (0.03)	1.73 (0.09)

Mean social engagement score was calculated based on the number of the items used. The summed score was divided by 8 for China and Russia, while it was divided by 9 for India, Ghana, South Africa and Mexico

Note: Weighted estimates

### Relationship between social engagement and tobacco and alcohol use and diet

The logistic regression results are presented in Appendix 2 (unadjusted model) and Table 3 (adjusted model). Appendix 2 presents the univariate logistic regression results examining the relationship between social engagement and six unhealthy lifestyle behaviors as well as subjective well-being across six countries. The results changed slightly after adjusting for sex, age group, marital status, education level, place of residence, income quintiles and working status (see Table 3). In China, higher level of social engagement was associated with higher risk of smoking, while in Ghana and South Africa, an inverse relationship was observed. For India, the relationship was no longer significant after controlling for the sociodemographic characteristics.

**Table 3:** Association between social engagement and unhealthy lifestyle behaviors as well as subjective well-being across six countries (adjusted logistic regression models)

	China OR (95% CI)	Russia OR (95% CI)	India OR (95% CI)	Ghana OR (95% CI)	South Africa OR (95% CI)	Mexico OR (95% CI)
Tobacco and alcohol use and diet						
Tobacco use (Ref: not current smokers)	1.19 (1.02–1.40)	0.79 (0.52–1.20)	0.96 (0.82–1.12)	0.81 (0.66–0.98)	0.58 (0.45–0.74)	1.20 (0.69–2.09)
Harmful drinking (Ref: < 14 standard drinks/week)	1.21 (0.94–1.55)	0.98 (0.44–2.21)	1.76 (0.90–3.43)	1.10 (0.86–1.42)	0.73 (0.40–1.33)	1.00 (0.37–2.73)
Low fruit and vegetable intake (Ref: ≥ 5 serves/day)	0.48 (0.35–0.65)	0.45 (0.30–0.68)	0.56 (0.45–0.70)	1.30 (1.13–1.51)	0.88 (0.69–1.12)	0.82 (0.48–1.42)
Activity-related behaviors						
Physical inactivity (Ref: ≥ 600 MET-minutes/week)	0.64 (0.47–0.86)	0.31 (0.19–0.50)	0.59 (0.49–0.72)	0.38 (0.32–0.45)	0.77 (0.60–0.97)	0.42 (0.29–0.60)
Prolonged sitting time (Ref: ≤ 7 hours/day)	0.87 (0.68–1.12)	1.40 (0.90–2.18)	0.99 (0.81–1.22)	0.70 (0.58–0.84)	0.40 (0.24–0.65)	0.58 (0.22–1.57)
Unhealthy sleep time (Ref: 7–9 hours/day)	0.73 (0.63–0.85)	0.86 (0.66–1.11)	0.84 (0.70–1.01)	0.74 (0.64–0.84)	1.15 (0.94–1.40)	–
Subjective well-being						
Perceived depression (Ref: not perceived depression)	0.80 (0.65–0.99)	0.53 (0.39–0.73)	0.88 (0.74–1.05)	1.13 (0.97–1.31)	1.05 (0.83–1.33)	0.61 (0.38–0.99)
Poor self-rated health (Ref: moderate and good self-rated health)	0.72 (0.58–0.89)	0.52 (0.32–0.86)	0.76 (0.63–0.91)	0.53 (0.45–0.63)	0.67 (0.49–0.93)	0.36 (0.15–0.83)
Low quality of life (Ref: high quality of life)	0.54 (0.44–0.67)	0.35 (0.22–0.56)	0.54 (0.44–0.66)	0.57 (0.49–0.66)	0.68 (0.52–0.89)	0.19 (0.07–0.50)

Adjusted for sex, age group, marital status, education level, place of residence, and income quintiles

Note: Weighted estimates

Social engagement was not associated with harmful drinking in any of these six countries. And only in Ghana, high social engagement had a positive relationship with low fruit and vegetable intake, while an inverse relationship was found in China, Russia, and India.

### Relationship between social engagement and activity-related behaviors

Social engagement was found to have an inverse relationship with physical inactivity across all six countries. It was also found that social engagement was inversely associated with prolonged sitting time in Ghana and South Africa, while no significant relationship in China, Russia, India, and Mexico. Social engagement was inversely associated with the risk of unhealthy sleep duration in China and Ghana only.

### Relationship between social engagement and subjective well-being

There was strong evidence for an association between high levels of social engagement and poor self-rated health and low quality of life in all six countries, as well as perceived depression (not significant in India, Ghana and South Africa) even after adjusting for the other sociodemographic variables.

## Discussion

This study is among the first to compare the relationships between social engagement and multiple lifestyle behaviors as well as subjective well-being in LMICs using nationally representative samples. Our study found consistent relationships between social engagement and activity-related behaviors as well as subjective well-being, while the relationships with tobacco and alcohol use and diet vary considerably across countries.

### Tobacco, alcohol use and diet

Our study showed that, in China, higher levels of social engagement were associated with higher risk of smoking, while an inverse association was found in Ghana and South Africa. The results from China are contrary to those found in other studies. Results from the longitudinal British Household Panel Survey (BHPS), showed that active social participation was positively associated with smoking cessation [11]. In a US Study, Samuel et al. also reported that emotional social support and neighborhood social cohesion were generally linked to lower smoking rates [7]. However, in the same paper, the authors pointed out that social support and social cohesion may be associated with higher smoking rate in groups with high rates of smoking, which is in line with previous research [25–27] and the current study. Past research has shown that the greater the social network size, the more likely a person is to smoke, especially in Asian cultures where collectivism is valued [28]. China consumes about 40% of the world’s cigarettes, and the prevalence of smoking remained high in men (54.0% prevalence for current-smoking) [29]. In many Asian countries [30], smoking with others is seen as a way to foster relationships between family members, peers, and business associates [31]. Tobacco can also be exchanged as “social currency” for social opportunities and benefits, which permeates every aspect of family life and wider social interactions [32, 33]. As there were no widespread anti-smoking public health program implemented in these countries, people view smoking in social activities as acceptable or even desirable, rather than a behavior to be reprimanded or punished [33]. This might explain the positive association between social engagement and smoking in China.

Social engagement was not found to be associated with alcohol consumption across the six LMICs, which is different from that reported in some HICs, where highest levels of alcohol consumption were noted [34]. Research in the United States [35] and Australia [14] found that social drinking was part of the social fabric among older peoples and retirees. Many participants in HICs emphasized the social nature of their alcohol use, as something to be enjoyed with others. During social activities, people may be less aware of how much they were drinking, and thus consume more than they would in non-social situations [14].

In our study, low levels of social engagement were associated with low fruit and vegetable consumption in China, Russia and India, but an inverse association was found in Ghana. This is a novel finding to our knowledge. A healthy diet is important to health and well-being at all stages in life; however, the determinants of dietary health change with age. Older adults are prone to developing an unhealthy dietary pattern for many reasons, including reduced mobility and/or fewer financial resources to spend on food [6, 7]. Moreover, socially isolated older adults are at a greater risk of poor dietary behavior because of the lack of social support [36]. Jones et al. found that getting out of the house and being active were effective in stimulating appetite [37], and eating with others has been shown to increase food intake by 60% in healthy older adults aged 65 and over [38]. Holmes also suggested that those who usually eat alone at home may substitute a cooked or hot meal with convenient food that can be easily accessed and prepared, such as sandwiches [38]. There was no evidence in the literature that explained why low social engagement was inversely associated with low fruit and vegetable intake in Ghana. One possible reason might be that when eating out, people usually have a high intake of meat products and beverage instead of fruit and vegetable [39, 40]. To fully understand this question, more culturally specific information about social eating behavior and the cultural context of food in Ghana is required.

### Activity-related behaviors

Our study showed that social engagement was inversely related to physical inactivity, prolonged sitting time and unhealthy sleep duration. These results are similar across the six countries and consistent with previous research. In the study by Samuel et al. [7], social support and neighborhood social cohesion were associated with achieving the recommended level of physical activity. Hiroyuki and colleagues [8] had similar findings among a Japanese sample, where men and women reporting higher social participation were less likely to be physically inactive. The possible mechanism could be that social engagement provides older people with more opportunities and social reasons to go outside to join physical activities [8].

According to ecological models [41], the physical and social environment in which people live are important determinants of sedentary behavior. In our study, we found that social engagement was inversely associated with prolonged sitting time in Ghana and South Africa, while no relationship was found in China, Russia, India and Mexico. Hiroyuki’s study [8] showed that higher levels of social participation was associated with less sedentary time, such as television watching, sitting around and listening or talking while sitting. But Van Holle et al. [10] found no evidence for the association between social isolation and talking with neighbors, with sedentary time. One possible explanation for the inconsistent results across countries might be the unmeasured domains of sedentary behaviors, for example mentally-active sedentary behavior (e.g. reading books). It is important to distinguish between different types of sedentary behaviors instead of considering them as one entity because social participation pattern may vary across domains of sedentary activities. Additionally, these results may also be partly influenced by age and working status, as well as whether the sitting time was self-reported or objectively measured, which requires further study.

Our study showed that people with higher levels of social engagement were less likely to be outside the optimal sleep range. This might be because a lack of social contacts in older people’s lives may result in the flattening of their circadian rhythm and reduced needs for sleep in the evening [42]. Thida et al. [43] found that having lower neighborhood social capital was associated with insufficient sleep among Japanese adults, particularly in the men. Tarja and colleagues [44] found that people with high levels of social support were more likely to have adequate duration of sleep. Such findings from observational studies have been confirmed by a cluster randomized trial by Joachim and colleagues among nursing home residents, where social activity sessions, including parlor games and group discussions, improved subjective sleep quality of the participants at both clinical and statistical significance levels [45].

### Subjective well-being

In our study, higher level of social engagement was consistently associated with less perceived depression, better self-rated health and higher quality of life. A Japanese longitudinal study showed that social engagement improves older people’s mental health, including depressive symptoms and psychological distress. Results from the 4^th^ National Household Health Survey [46] also found that social contacts were positively associated with quality of life among Chinese older adults in urban areas. Snorri [47] observed that social network size and contact frequency were positively and independently related to future subjective well-being in English adults aged 50 and older. Findings from the National Social Life, Health, and Aging Project [48] among older Americans confirmed that networks with a wider range of social ties were related to better well-being, independent of demographic and health characteristics.

Social engagement could increase people’s social networks, which leads to attachment, esteem, social approval, belongingness, social identity and increasing access to social support [49]. In particular, social network could provide access to functional support and assistance from family members, neighbors and friends. This type of support is important and have been shown to be related to older adults’ improved sense of control, enhanced quality of life and wellbeing [47].

Overall, this international study found high level of social engagement as a consistent correlate of health in some LMICs. Regarding subjective well-being, social engagement appears to be protective of perceived depression, poor self-rated health and low quality of life. However, for lifestyle risk behaviors, the associations varied by the outcome and the country, possibly as a result of the different ways people socialize. It is also important to note that as a cross-sectional study, the relationships between social engagement, lifestyle behaviors and subjective well-being could be bidirectional, that is either social engagement promotes healthier behaviors and mental health or healthy behaviors and subjective well-being lead to higher levels of social engagement. Additionally, across countries, social engagement was associated with almost all outcomes in Ghana and China, but less so in other countries. This suggests that the relationship between social engagement and lifestyle behavior is complicated and culture-specific. Future research should focus on examining various cultural elements when measuring both social engagement and health behaviors.

### Strengths and limitations

Strengths of this study include comparable data from six LMICs with nationally representative samples. This paper is among the first to examine a broad range of lifestyle behaviors and subjective well-being, in relation to social engagement with a large sample size across LMICs. However, some limitations should be noted. First, a cross-sectional design limits causal inferences. Second, self-reported measures are subject to reporting bias. Third, some unknown confounders, such as BMI and existing chronic disease, were not collected and therefore could not be adjusted for. Finally, it is arguable that there might be differences in the interpretation of social engagement questions across countries because of the inherent differences in cultural and social norms. Thus, these societal aspects are worthy of further exploration to help explain our findings.

## Conclusion

In conclusion, our study extends prior research by exploring the associations between social engagement and multiple lifestyle behaviors as well as subjective well-being in LMICs. Although the associations were mixed across countries, findings are generally consistent in supporting the notion that higher levels of social engagement may promote activity-related behaviors and subjective well-being. Our findings highlight the need for policy makers to consider how social engagement can be incorporated in preventive health, with a potential range of benefits to the health of older populations. Interventions at individual and community levels should encourage and facilitate older adults to become more socially engaged within specific cultural context.

## Data Availability

The datasets used and analyzed during this study are publicly available on the official website, https://www.who.int/healthinfo/sage/cohorts/en/.

## Appendix

**Table 4:** Social engagement items and Cronbach’s α coefficient for each country

How often in the last 12 months have you …						
Q1 … attended any public meeting in which there was discussion of local or school affairs?						
Q2 … met personally with someone you consider to be a community leader?						
Q3 … attended any group, club, society, union or organizational meeting?						
Q4 … worked with other people in your neighbourhood to fix or improve something?						
Q5 … had friends over to your home?						
Q6 … been in the home of someone who lives in a different neighbourhood than you do or had them in your home?						
Q7 … socialized with co-workers outside of work?						
Q8 … attended religious services (not including weddings and funerals)?						
Q9 … gotten out of the house/your dwelling to attend social meetings, activities, programs or events or to visit friends or relatives?						
	China*	Russia*	India	Ghana	South Africa	Mexico
Cronbach’s α	0.63	0.73	0.75	0.81	0.75	0.72

* Cronbach’s alpha coefficient based on 8 items (excluding Q8)

**Table 5:** Association between social engagement and unhealthy lifestyle behaviors as well as subjective well-being across six countries (unadjusted logistic regression models)

	China OR (95% CI)	Russia OR (95% CI)	India OR (95% CI)	Ghana OR (95% CI)	South Africa OR (95% CI)	Mexico OR (95% CI)
Tobacco and alcohol use and diet						
Tobacco use (Ref: not current smokers)	1.45 (1.25–1.69)	1.20 (0.84–1.72)	1.36 (1.15–1.62)	0.81 (0.72–0.93)	0.63 (0.50–0.80)	1.26 (0.64–2.48)
Harmful drinking (Ref: < 14 standard drinks/week)	1.75 (1.43–2.15)	1.03 (0.56–1.92)	2.58 (1.55–4.29)	1.53 (1.20–1.97)	0.80 (0.45–1.43)	0.43 (0.07–2.56)
Low fruit and vegetable intake (Ref: ≥ 5 serves/day)	0.41 (0.30–0.55)	0.49 (0.36–0.66)	0.42 (0.35–0.50)	1.22 (1.08–1.39)	0.84 (0.66–1.06)	0.75 (0.35–1.59)
Activity-related behaviors						
Physical inactivity (Ref: ≥ 600 MET-minutes/week)	0.55 (0.41–0.72)	0.15 (0.09–0.27)	0.43 (0.36–0.51)	0.32 (0.28–0.37)	0.67 (0.53–0.84)	0.44 (0.27–0.71)
Prolonged sitting time (Ref: ≤ 7 hours/day)	0.79 (0.63–0.99)	1.52 (0.94–2.46)	0.95 (0.79–1.14)	0.79 (0.67–0.92)	0.42 (0.23–0.75)	0.64 (0.29–1.39)
Unhealthy sleep time (Ref: 7–9 hours/day)	0.65 (0.56–0.76)	0.65 (0.48–0.88)	0.89 (0.73–1.09)	0.73 (0.64–0.83)	1.00 (0.82–1.22)	–
Subjective well-being						
Perceived depression (Ref: not perceived depression)	0.72 (0.59–0.89)	0.36 (0.26–0.51)	0.68 (0.59–0.79)	0.98 (0.87–1.12)	1.04 (0.84–1.30)	0.58 (0.38–0.88)
Poor self-rated health (Ref: moderate and good self-rated health)	0.64 (0.53–0.76)	0.22 (0.13–0.37)	0.52 (0.43–0.62)	0.41 (0.35–0.48)	0.64 (0.49–0.85)	0.38 (0.16–0.91)
Low quality of life (Ref: high quality of life)	0.48 (0.39–0.59)	0.22 (0.14–0.35)	0.36 (0.30–0.45)	0.47 (0.41–0.54)	0.68 (0.54–0.86)	0.27 (0.11–0.69)

Note: Weighted estimates

## Abbreviations

GPAQ: Global Physical Activity Questionnaire
HICs: High Income Countries
LMICs: Low- to Middle-Income Countries
SAGE: Study on Global Ageing and Adult Health
WHO: World Health Organization
WHOQOL-BREF: World Health Organization Quality of Life Instrument-Abbreviated Version
